# Shocker—A Molecular Dynamics Protocol and Tool for Accelerating and Analyzing the Effects of Osmotic Shocks

**DOI:** 10.1021/acs.jctc.3c00961

**Published:** 2023-12-18

**Authors:** Marco P. A. van Tilburg, Siewert J. Marrink, Melanie König, Fabian Grünewald

**Affiliations:** † Groningen Biomolecular Sciences and Biotechnology Institute and Zernike Institute for Advanced Materials, University of Groningen, 9747 AG Groningen, The Netherlands; ‡ Heidelberg Institute for Theoretical Studies (HITS), Schloss-Wolfsbrunnenweg 35, 69118 Heidelberg, Germany

## Abstract

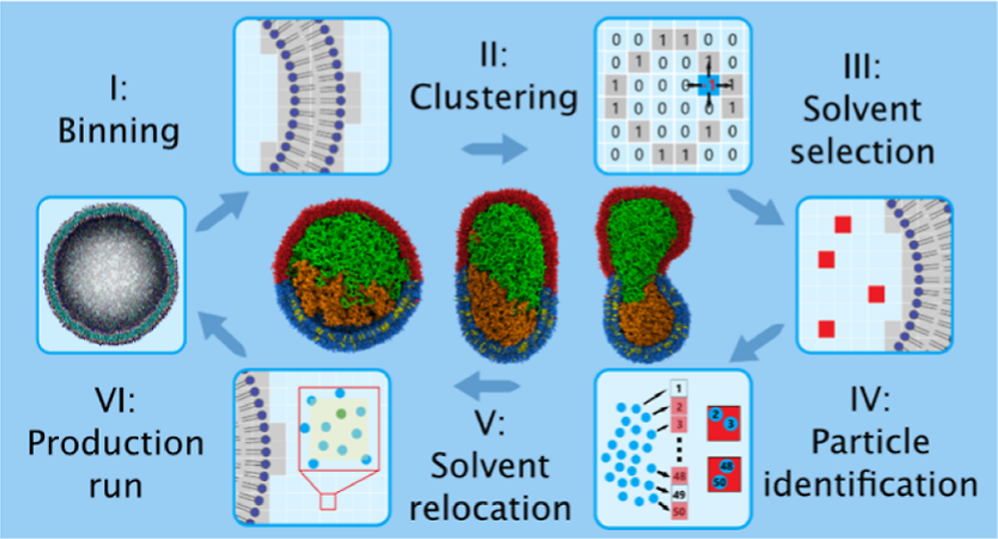

The process of osmosis, a fundamental phenomenon in life, drives water through a semipermeable membrane in response to a solute concentration gradient across this membrane. In vitro, osmotic shocks are often used to drive shape changes in lipid vesicles, for instance, to study fission events in the context of artificial cells. While experimental techniques provide a macroscopic picture of large-scale membrane remodeling processes, molecular dynamics (MD) simulations are a powerful tool to study membrane deformations at the molecular level. However, simulating an osmotic shock is a time-consuming process due to slow water diffusion across the membrane, making it practically impossible to examine its effects in classic MD simulations. In this article, we present Shocker, a Python-based MD tool for simulating the effects of an osmotic shock by selecting and relocating water particles across a membrane over the course of several pumping cycles. Although this method is primarily aimed at efficiently simulating volume changes in vesicles, it can also handle membrane tubes and double bilayer systems. Additionally, Shocker is force field-independent and compatible with both coarse-grained and all-atom systems. We demonstrate that our tool is applicable to simulate both hypertonic and hypotonic osmotic shocks for a range of vesicular and bilamellar setups, including complex multicomponent systems containing membrane proteins or crowded internal solutions.

## Introduction

Osmosis is one of the most fundamental phenomena in life which occurs when a solute concentration gradient exists across a semipermeable membrane, e.g., the lipid membrane of a biological cell. Due to the selective permeability of the membrane, solvent molecules diffuse along this osmotic gradient from a region of low solute concentration to a region of high solute concentration, thereby equalizing the chemical potential in the two compartments. Osmosis is a vital process for a wide variety of organisms. In plant cells, for example, osmotic pressure leads to water influx into cells, which increases the internal pressure, the so-called turgor pressure, against the cell wall. The turgor pressure controls the cell size, geometry, and rigidity, which are required to maintain their cellular stiffness.^1^ Bacteria, on the other hand, developed mechanisms to resist osmotic fluctuations, enabling them to survive in a wide range of environments and solute concentrations. By accumulating or synthesizing solute molecules, they maintain an internal solute concentration equivalent to that of the environment.^2^ To cope with sudden solute concentration changes, bacteria use mechano-sensitive water channels, such as MscL and MscS,^3^ acting as an osmotic pressure release valve.

Water movement across the plasma membranes of cells causes them to deflate or inflate, which is usually accompanied by shape changes. In vivo, membrane shape remodeling plays an active role in the regulation of many cellular processes, including cell fission.^4,5^ Before cell fission can occur, an excess of membrane area is needed, which is obtained by lipid production and cell growth.^6^ The excess membrane is used to reshape the spherical cell into a dumbbell, a crucial intermediate for cell division events.^7^ In experimental reconstitution studies, giant unilamellar vesicles (GUVs) are commonly used as (artificial) cell models.^8−12^ The required excess membrane in these studies is obtained by decreasing the volume of the vesicle through a hypertonic osmotic shock induced by a sudden change in the exterior solute concentration. To drive membrane fission, many cell and synthetic biology studies combined this approach of osmotic deflation with (i) encapsulation of scaffolding proteins or protein machinery^13,14^ (ii) introduction of spontaneous curvature by protein crowding^15^ or changes in pH;^16^ (iii) insertion of lipophilic compounds;^17,18^ (iv) induction of liquid–liquid phase separation;^19,20^ (v) temperature cycling;^21^ or (vi) microfluidic devices to apply a mechanical force.^22^

Although the mentioned experimental studies led to promising fission results, the exact mechanism remains unknown since these fission events happen on a too short time scale to observe the process directly with sufficient detail. To overcome these shortcomings, computer simulations can be used to gain near-to-atomistic-level resolution of membrane deformations at nano- to microsecond time scales.^23^ Especially, molecular dynamics (MD) simulations at the coarse-grained (CG) level have proven to be a valuable tool in studying vesicle shape transformations and fission.^24−28^ Ghosh et al.,^24^ for example, constructed a range of spherical CG vesicles with different initial volumes and varying lipid ratios in the inner and outer leaflets to study differences in their shape deformations during volume reduction. They concluded that, upon deflation, the initially spherical vesicles transformed into oblates, prolates, and dumbbells, depending on the lipid ratio between the leaflets. In another approach, Markvoort and Marrink^26^ simulated membrane fission pathways by distributing two nonmiscible lipid types between the inner and outer leaflet, starting from an already deformed vesicle. When both lipid types were distributed equally in the two leaflets, they observed pronounced phase separation. The resulting line tension caused a decrease in the neck radius of the initially dumbbell-shaped vesicle. Eventually, the authors observed asymmetric fission, i.e., the resulting daughter vesicles had a different lipid composition than the mother vesicles. However, when the two lipid types were distributed asymmetrically so that each leaflet contained only one type of lipid, an increase in spontaneous curvature led to symmetric fission.

The studies mentioned above show that MD simulations are suitable for examining the shape behavior of lipid vesicles during volume changes. However, to induce shape changes, i.e., change the initial area-to-volume ratio and generate an excess in membrane area, they rely on pre-deflated or pre-deformed vesicles instead of simulating an explicit osmotic shock. The reason is that naively simulating an osmotic shock by letting solvent molecules diffuse along a concentration gradient through a membrane is too slow to induce any shape changes on typically accessible time scales in MD simulations. For example, Hong et al.^29^ constructed a small atomistic bilayer consisting of 210 lipids and measured the water permeability of this membrane. After a 2 μs simulation, 50 permeation events were counted, which is equivalent to 2000 permeation events in a vesicle with a radius of 16 nm. Considering that such a vesicle contains around 150,000 water particles, in theory, a 75 μs simulation would be needed to yield a volume reduction of 50%. This calculation, however, does not account for any counter flux or the steady decrease in osmotic pressure upon water efflux. Therefore, realistic simulation times are likely in the range of several hundred microseconds.

While active water removal from the vesicle interior during the initial setup is a simple and suitable approach to avoid time-consuming osmotic shock and yield general biophysical insights into their shape behavior, this approach allows very little control over obtaining specific shapes and deformation pathways. Considering the inherent slowness of osmotic shocks in vivo, removing a substantial amount of water instantaneously can alter the membrane dynamics, resulting in nonphysical shape deformation pathways. As demonstrated by Yuan et al.,^30^ when applying different volume-change rates to a mesoscopic model, different deformation pathways could be observed. At a slow rate, the membrane had sufficient time to relax the internal stress, resulting in prolate and dumbbell-shaped vesicles. On the other hand, a fast volume-change rate led to the accumulation of membrane stress, resulting in predominately biconcave oblate (discocyte)-shaped vesicles. The use of pre-deformed vesicles as a starting point for further shape deformations could alleviate this problem; however, setting up realistic membrane shapes with a complex composition is currently still a nontrivial task.^23^

Another method to mimic explicit osmotic shocks in MD simulations relies on the iterative removal of solvent particles from the system. Such an approach is more promising, as it does not require the complex setup of realistic membrane shapes and inefficient passive solvent diffusion. While previous studies were able to generate osmotically driven shape changes in simple lipid vesicles consistent with experimental studies,^31,32^ no ready-to-use protocol applicable to more complex systems has been published. Thus, a general protocol for applying an explicit osmotic shock would offer so far unexplored opportunities, e.g., in studying the curvature sensing and remodeling behavior of proteins and lipids.

To accelerate osmosis in simulations and allow better control over the produced shapes, we developed a new MD protocol that is implemented into the Shocker Python package. This protocol mimics the effects of water efflux (influx) during a hypertonic (hypotonic) shock by relocating solvent particles from the inner to outer compartments (or vice versa). Although other protocols exist for removing or inserting solvent molecules,^33,34^ there is, to our knowledge, no ready-to-go protocol for osmotic shocks which is also compatible with the Martini CG force field. In addition to the protocol, we also implemented common analysis metrics to quantify vesicle shapes and shape changes such as the reduced volume and the reduced area difference, which are explained in detail in the Supporting Information Methods. Shocker utilized the Gromacs simulation engine to run the actual MD simulations. Other MD engines can, in principle, be used but are currently not supported.

The remainder of this work is organized as follows: First, we describe the implementation of the solvent relocation scheme for mimicking an osmotic shock exemplified by a hypertonic shock applied to a vesicle. Afterward, we consider five application systems: (1) hypertonic shocking of a simple vesicle, (2) hypotonic shocking of a simple vesicle, (3) hypertonic shocking of a crowded vesicle, (4) hypertonic shocking of a vesicle in the presence of proteins, and (5) hypertonic shocking of an atomistic double bilayer system. These systems serve to validate that the Shocker protocol can be applied to a wide range of biologically relevant systems and produce shape changes that are physically realistic and consistent with trends described in the literature, if available. Finally, we benchmark the performance and discuss limitations, as well as future improvements.

## Algorithm

**Figure 1:**
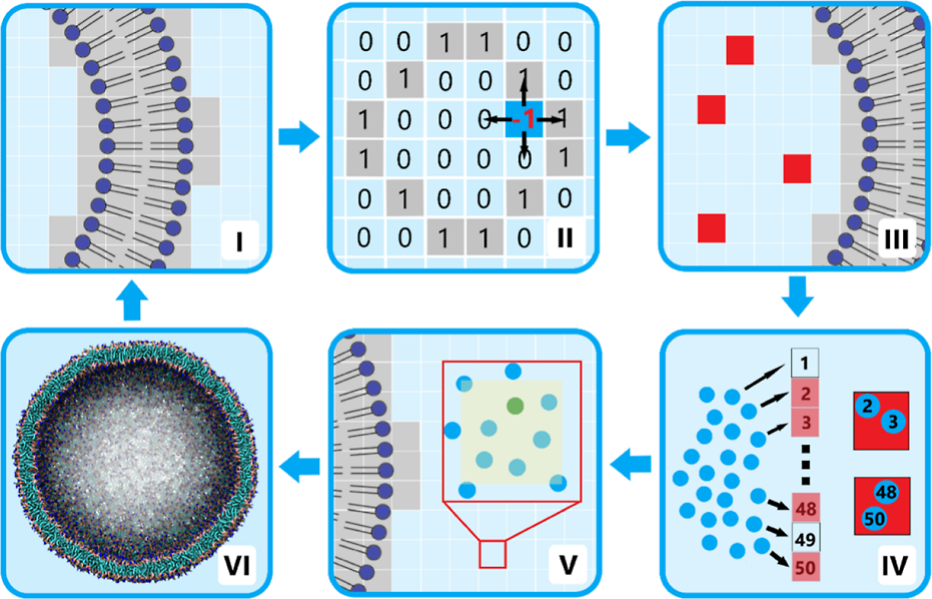
Workflow overview of one pumping cycle. (I) Initially, the original system is converted to a system of membrane bins (gray) and solvent bins (blue). (II) Subsequently, the solute bins are clustered using a graph-based clustering algorithm. (III) Solvent bins are randomly selected from the inner (outer) compartment for a hypertonic (hypotonic) shock, and (IV) exact positions of solvent molecules in these bins are calculated. (V) One solvent molecule per bin is relocated to an “empty” position in the other compartment, after which a production run is initiated (VI).

To simulate the continuous efflux (influx) of solvent molecules during an osmotic shock, the simulation is divided into several pumping cycles. A pumping cycle includes the identification of solvent particles, the relocation of them into target bins, and the updating of the topology. In this section, we will provide a detailed description of the workflow of this cycle and discuss the underlying algorithms in six steps, which are visualized in Figure 1.

In the following, a vesicle system is used as an example, but the code is also applicable to other systems that feature an enclosed water compartment. In order to set up and initiate a pumping simulation, the user should provide the structure file (GRO) of a fully energy-minimized and equilibrated vesicle. Besides, a topology file (TOP) and an index file (NDX) should be provided, where the index file should at least discern membrane and solvent particle groups. The online tutorials, deposited on our GitHub page (https://github.com/marrink-lab/shocker) describe more practical details concerning the input files and other requirements.

### Step I: Binning the System

The pumping cycle starts with binning the system while conserving the system dimensions (Figure 1, Step I), which drastically speeds up calculations compared to when considering each particle individually. The bin-edge size is adjustable; however, we recommend using the default bin-edge size of 1.3 nm, which works well for both AA and CG systems. To distinguish between membrane and solvent bins, the provided index file is used to translate the positions of all membrane particles to the bins in which they reside, which receive the value 1. Subsequently, all remaining bins are set to 0 and are automatically part of the solvent group. Important to note here is that all structures residing in the membrane, such as proteins or cholesterol, should be in the same index group as the lipids to avoid a noncontiguous bilayer bin system during the subsequent bin clustering process. The solvent group must solely contain the solvent particles that are supposed to be relocated. Ions, soluble proteins, polymers, or any other molecule residing in the interior or exterior of the vesicle are omitted during the binning process.

### Step II: Clustering of the Membrane and Solvent Bins

To discern the inner and outer solvent compartments, the solvent bins are clustered using a distance-based clustering algorithm in which only adjacent bins are considered (Figure 1, Step II).^35^ The algorithm starts in a bin with a value of 0, indicating a solvent bin. From this position, it considers the values of the six adjacent bins, of which the position is added to the cluster if it turns out to be a zero. In Figure 1, Step II, these would be the bins below and left of the current bin. Subsequently, the current bin receives a value of −1, indicating that this bin has already been visited, after which the algorithm proceeds to the next zero. If eventually no adjacent zeros are available (the zero list is empty), the algorithm jumps to a new, not processed zero, for as long as there are zeros in the box. The clustering takes the periodic boundaries into account, such that clusters split over PBC are correctly identified as single clusters. We note that the clustering procedure is currently limited to rectangular PBC conditions. The outer solvent cluster (i.e., bulk solvent) is then identified by counting the number of bins located in the corners of the simulation box within the solvent cluster. The cluster that contains the largest number of corner bins is taken to be the outer solvent cluster. This assignment strategy breaks down when the vesicle crosses one of the box corners under the PBC. Thus, we recommend starting with a box-centered vesicle.

The default bin-edge size is 1.3 nm. Working with a smaller bin size increases precision, as fewer solvent particles positioned close to the membrane surface are included in the membrane bin section. The downside of a smaller bin size is the increased probability of creating a noncontiguous membrane bin structure, ultimately causing the clustering algorithm to fail as all solvent molecules will form a single compartment. Using a smaller bin size also increases the total number of bins and, therefore, the overall computation time.

### Step III: Solvent Selection

The first step in solvent relocation is identifying the solvent particles that need to be relocated. Since the workflow example considers a hypertonic shock, the solvent is moved from the inner to the outer compartment. Bins are randomly selected from the clustered inner solvent compartment (Figure 1, Step III). If a selected bin contains other molecules than just the solvent, then it will be discarded and another bin will be selected. This ensures that the hydration shell of, for example, soluble proteins and polymers remains untouched. Only if no solvent-only bins can be identified will those bins be considered further. From each of the selected bins, only one solvent molecule is chosen to be relocated to ensure that the solvent is removed from areas that are well dispersed across the whole inner compartment. The number of solvent molecules to be relocated (and hence the number of bins considered) is specified by the user.

### Step IV: Particle Identification

First, all water particles are binned, yielding an *N* × 3 integer array of bin addresses. Subsequently, to identify the water particles residing in the previously selected bins, we find all matches to the selected bin addresses in the *N* × 3 array (Figure 1, Step IV). The resulting array of indices is then converted to the global indices, which identify the solvent particles in the positions array.

### Step V: Solvent Relocation

After solvent particles were selected, new positions had to be found in the target bins. Relocating particles by simply choosing random coordinates usually causes overlap between particles, leading to numerical instabilities. Shocker’s relocating algorithm always tries to find the most suitable locations by taking the presence of other structures and particles into account (Figure 1, Step V), thereby avoiding significant overlap.

The water placement starts with selecting a suitable bin for relocation, which ideally contains only solvent molecules. In cases where such bins are not available, a bin containing the smallest number of solute particles is selected. Subsequently, within this bin, a spot is identified with the largest distance from the nearest neighboring particles by performing trial insertions. To avoid overlap with particles residing in neighboring bins, this position must be within 0.3 nm of the edges of the selected bin.

### Step VI: Production MD Run and Steady State

Once the desired number of solvent particles has been relocated, a production run is initiated, which completes the pumping cycle (Figure 1, Step VI). The length of these production runs is defined by the user and greatly depends on the type of system. For example, for a vesicle with a radius of 15 nm, we used a production run of 2 ns between each pumping cycle.

Besides hypotonic and hypertonic osmotic shocks, Shocker can perform a steady-state run in which the volume of a vesicle, regardless of the shape, is kept constant. To this end, the number of enclosed water particles at the start of the steady-state run is calculated and compared to the current number after each cycle, which typically lasts 2 ns. Considering that a significant fraction of solvent molecules can reside in the interior of the membrane, they might belong to the vesicle interior in one cycle and to the exterior in the next one due to the rigorous clustering procedure in Step II. This means that the target number of solvent molecules can never be precisely determined. Consequently, translocating the exact difference in solvent molecules could lead to overcompensation and, thus, further membrane deformation instead of a steady-state simulation. Therefore, the interior volume is averaged over 10 cycles, which means that the volume is adjusted only after 20 ns.

Usually, the osmotic shock simulation runs until the desired number of pumping cycles is reached. The only cases in which solvent pumping is completely terminated are when no solvent molecules are available for pumping or when the membrane ruptures. However, the user can also define a target interior volume or solute concentration. Once this target is reached, Shocker switches into steady-state mode, and further pumping cycles are initialized only when the interior volume changes. To this end, Shocker monitors the solute concentration and interior volume if these options are switched on by the user. A detailed description of how these properties are obtained is given in the Supporting Information Methods.

## Results

In the following, we apply an osmotic shock to five different example systems to validate Shocker’s algorithms. First, we simulated a simple CG vesicle composed of palmitoyloleoylphosphatidylcholine (POPC) lipids using the Martini 3 force field.^36^ We compare the effects of different pumping rates on the deformation pathways during a hypertonic osmotic shock. Second, we performed a hypotonic osmotic shock on a large POPC vesicle until the vesicle ruptured. Third, we simulated a more complex CG vesicle system composed of a phase-separating lipid mixture (dipalmitoylphosphatidylcholine (DPPC), dioctadecatrienoylphosphatidylcholine (DFPC), and cholesterol) that also contained a phase-separated polymer mixture (dextran and PEG) in the vesicle interior. Fourth, we studied a large CG POPC vesicle with a high concentration of aquaporin, a transmembrane protein. Lastly, to show Shocker’s compatibility with AA force fields, we also performed a pumping simulation on a double bilayer POPC system using the CHARMM36 force field.^37^ More details about the simulation setups are provided in the Methods section and Table S1.

### Pumping Rate Influences Vesicle Shape Changes

**Figure 2:**
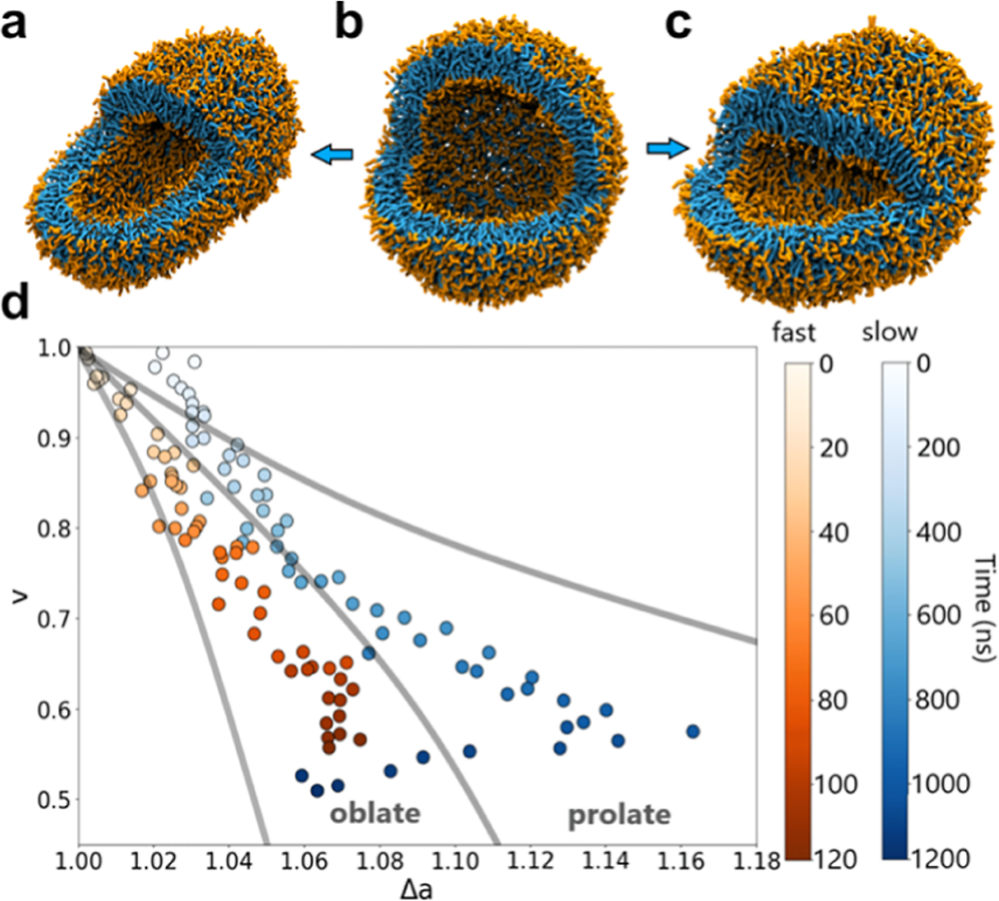
Effect of pumping rate during hypertonic osmotic shock. The snapshots in (a–c) show the shape deformation of the initially spherical vesicle (b) after 40% volume reduction using a slow pumping rate of 0.1% (a) and a fast pumping rate of 1% (c). Water molecules are omitted for clarity. (d) Each dot in the shape diagram (relative volume vs reduced area difference) represents the vesicle shape after a single pumping cycle. The color indicates the temporal evolution of the two pumping rates (white to dark red/blue). The oblate and prolate regions are indicated according to an experimental study by Käs and Sackmann.^38^

In the first test case, we used Shocker to perform a hypertonic shock on a simple POPC vesicle with a radius of 7.5 nm filled with water molecules (Figure 2b). We examine the effect of different water efflux rates, which have been shown previously to result in different shape remodeling pathways.^30^ Here, we used pumping rates of 0.1% (slow) and 1% (fast) of the initial vesicle volume, corresponding to the translocation of 10 and 100 water molecules per 2 ns, respectively.

Starting from a spherical vesicle (Figure 2b), we observe vesicle deformations toward prolate-shaped (Figure 2a) and oblate-shaped (Figure 2c) vesicles when pumping with a rate of 0.1 and 1%, respectively. To monitor shape changes over time, we calculated the reduced area difference Δ*a* and the reduced volume v of the vesicle after each pumping cycle. Since every possible vesicle shape has its unique *v*/Δ*a* ratio, the shape change over time can easily be visualized in a scatterplot (Figure 2d), where each dot represents the vesicle shape after one pumping cycle along the entire length of the trajectory. The isochores in Figure 2d defining oblate and prolate regions originate from an experimental study by Käs and Sackmann.^38^ For the faster pumping rate (red), the spherical vesicle turns into an oblate one within a couple of pumping cycles (Figure 2c). On the contrary, slow water relocation (blue) preferably leads to prolate vesicles (Figure 2a). However, removing more than 40% of the initial spherical volume causes a sudden change toward an oblate shape, comparable to the shape obtained with a faster pumping rate. In a 5 μs steady-state simulation (Figure S1), we observe that the shape of the vesicle that was subjected to the faster pumping rate shows a significant shift over time. The vesicle obtained from the slow-pumping simulation, on the other hand, exhibited a rather constant area difference, indicating that the fast pumping resulted in an accumulation of membrane stress that was relaxed during the steady-state simulation.

### Exploding Vesicle

Next, we simulate a hypotonic shock, i.e., water is translocated from the exterior to the interior of a vesicle, causing it to expand and eventually to rupture. Here, we validate whether Shocker is still able to relocate water particles to the inner compartment when the internal pressure rises. To this end, we created a large simulation box of 60 nm edge length containing a POPC vesicle with an initial radius of approximately 15 nm. To make the system more realistic and add another level of difficulty for the solvent insert process, we also added 0.15 M NaCl. For this simulation, a pumping rate of 200 particles every 2 ns was used, corresponding to 0.2% of the initial vesicle volume. After each pumping cycle, a short equilibration run of 20 ps with a time step of 2 fs is executed since the rise of internal pressure causes overlap of particles during the insertion step.

**Figure 3:**
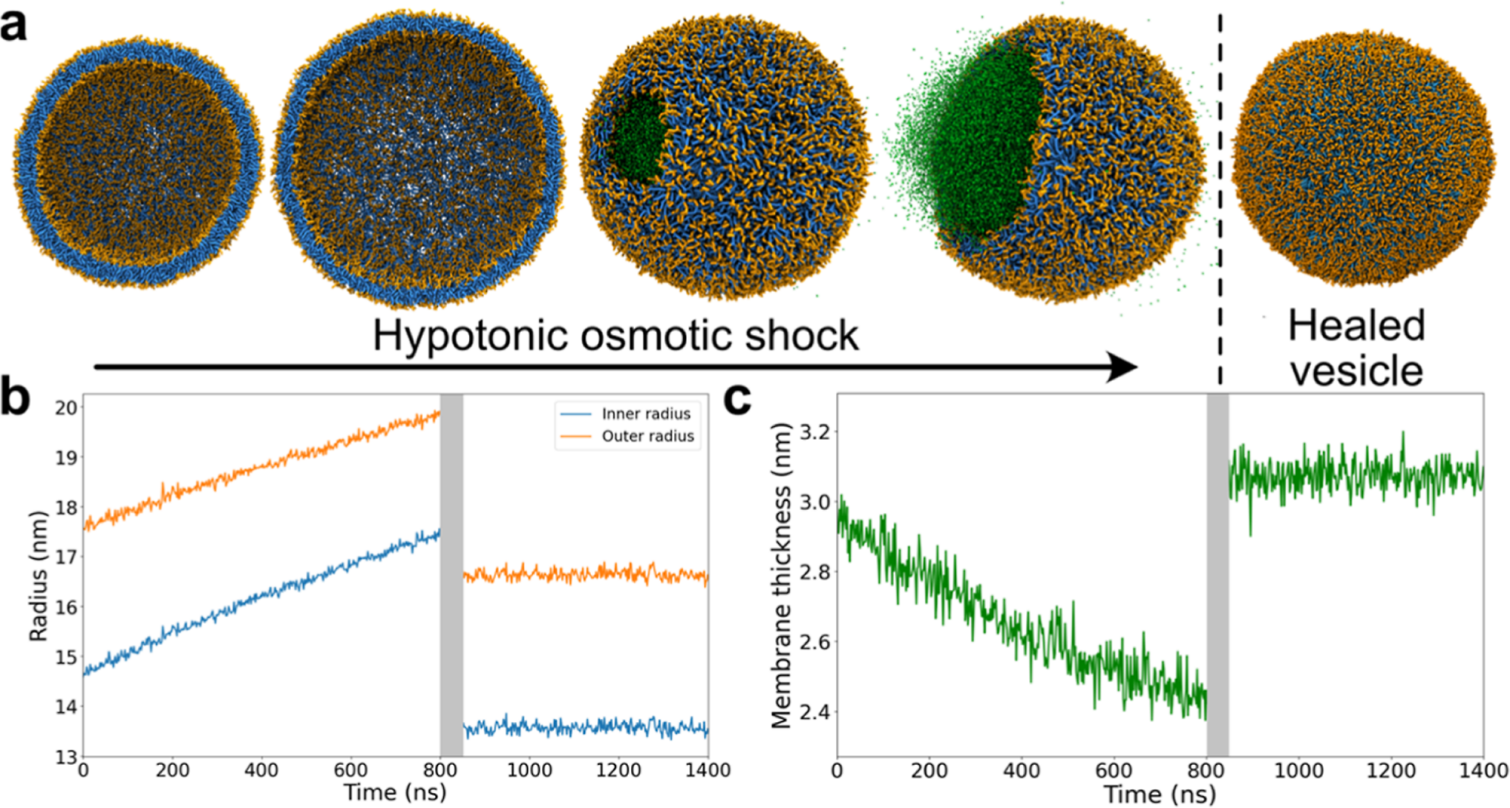
Hypotonic osmotic shock. (a) Snapshots of the stages of a hypotonic shock. The initial stage is an approximately tensionless vesicle filled with water and ions (omitted for clarity). Subsequently, Shocker pumps water from the exterior to the interior compartment, thereby increasing the internal pressure, which leads to thinning of the stressed membrane. Eventually, a pore is formed in the membrane due to the pressure inside. This pore expands fast, releasing excess water (green) and membrane stress. Finally, the vesicle undergoes a rapid healing process and returns to a closed state. The changes in (b) radius of the outer and inner leaflets and (c) membrane thickness are shown as a function of simulation time. The gray area in parts (b,c) indicates the presence of a pore.

To quantify the hypotonic shock, vesicle radius and membrane thickness were calculated after each pumping cycle. Hereby, the thickness is measured as the distance between the glycerol linkers of the lipids. Figure 3a and Movie S1 show that the continuous insertion of water molecules leads to the swelling of the vesicle. The accompanied rise in internal pressure causes the membrane to expand before it finally ruptures, which is in line with the results of an earlier study.^39^ During the pumping process, the vesicle volume grows, causing a steady increase in the radius of the outer and inner leaflets by 2 and 2.5 nm, respectively (Figure 3b). This difference can be explained by the decreasing membrane thickness, as shown in Figure 3c. At the rupture point, the vesicle volume increased to approximately 160% of its original volume. If a pore is present at the beginning of a pumping cycle, Shocker detects the noncontiguous membrane structure and switches to a regular (no-pumping) simulation with a default duration of 500 ns.

During a rapid healing period of less than 50 ns, as indicated by a gray bar in Figure 3b,c, the excess water particles are released, and the membrane returns to a closed state. At the end of the simulation, both the radii and the volume of the vesicle are significantly smaller than those of the initial vesicle. Also, the final membrane thickness is slightly larger than the initial thickness. Comparing the number of lipids per leaflet at the beginning of the simulation to the state after the pore closed, we find that the number asymmetry decreased by 8%. This means that 123 POPC lipids, which were originally located in the outer leaflet, are now part of the inner leaflet. Although the pore was present for only a few nanoseconds, the large rim (Figure 3a) allowed a fast exchange of lipids between the two leaflets.

### Deformation of a Phase-Separated, Polymer-Filled Vesicle

**Figure 4:**
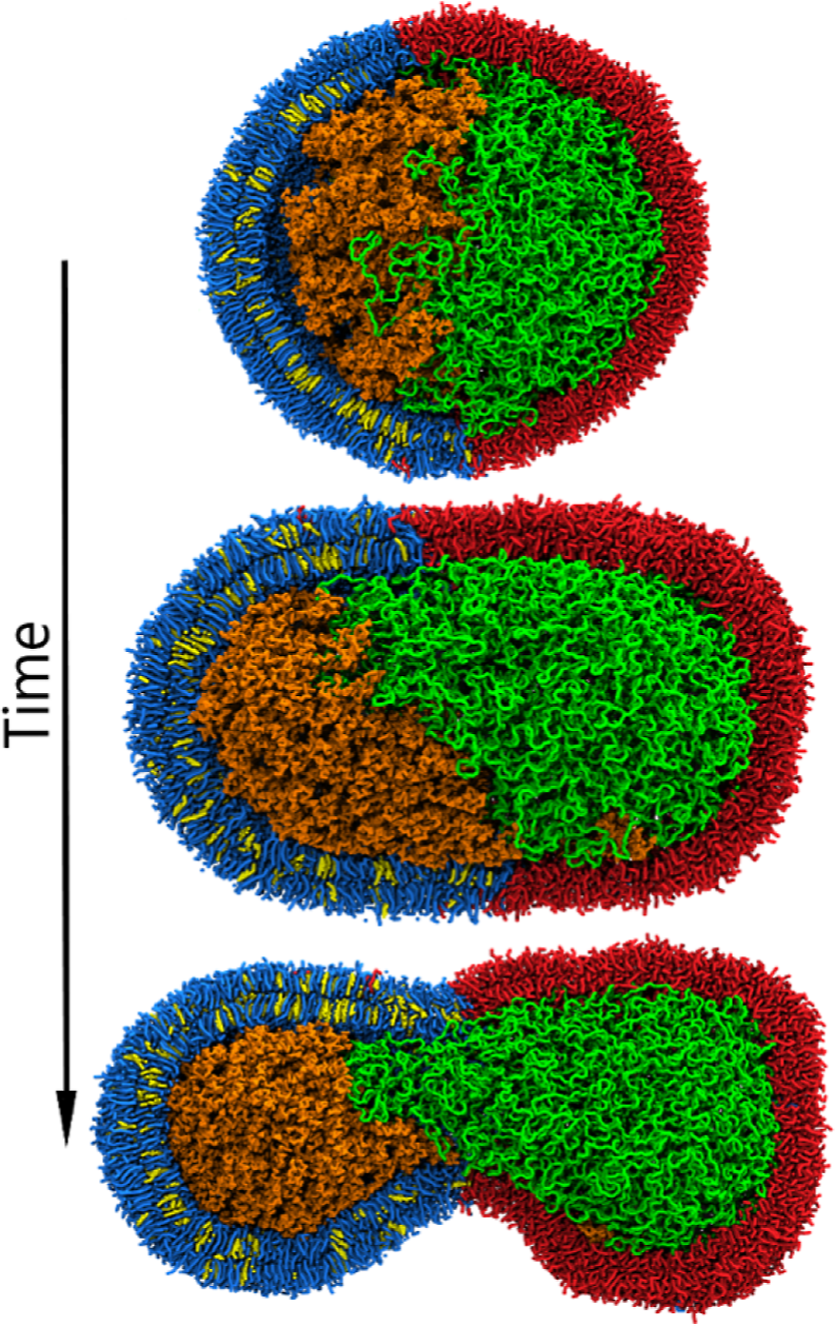
Hypertonic osmotic shock applied to a polymer-filled vesicle. The initial vesicle (top) consists of a phase-separated DPPC/CHOL/DFPC membrane depicted in blue, yellow, and red, respectively, and filled with dextran and PEG (orange and green). During a hypertonic osmotic shock, the vesicle first elongates and forms a prolate (middle) and eventually ends up with a dumbbell shape (bottom), with the neck being located at the interface of the two lipid domains.

In the third test case, we apply a hypertonic osmotic shock to a phase-separated vesicle with a crowded, polymer-filled inner compartment. We constructed a spherical CG vesicle with an initial radius of 15 nm and a membrane composed of three different lipid types, DPPC/CHOL/DFPC in a 0.3/0.2/0.5 ratio. The membrane was set up in an already phase-separated state, as can be seen in Figure 4 (top), with the liquid-ordered (L_o_) domain, containing the fully saturated DPPC lipids and CHOL, on the left and the liquid-disordered (L_d_) domain, containing the polyunsaturated DFPC lipids, on the right. Subsequently, the vesicle interior was filled with dextran and poly(ethylene glycol) (PEG), which are known to exhibit liquid–liquid phase separation. The dextran and PEG phases are aligned with the L_o_ and L_d_ domains, respectively. This setup roughly resembles the experimental setup presented by Andes-Koback and Keating,^19^ who studied asymmetric cell division by applying an osmotic shock to phase-separated and crowded lipid vesicles. As in the previous example, we used a pumping rate of 200 water particles every 2 ns, relocating approximately 0.2% of the initial vesicle solvent volume in each pumping cycle. After each pumping cycle, a short equilibration run of 20 ps with a time step of 2 fs was executed.

The deformation of the initially spherical vesicle over the course of 185 pumping cycles is shown in Figure 4 and Movie S2. As the volume decreases, the vesicle starts to elongate and eventually deform into a dumbbell shape due to the line tension between the two lipid phases. Simultaneously, the phase-separated polymer mixture in the vesicle interior minimizes the interfacial area, further facilitating the formation of a dumbbell-shaped vesicle. This finding is in line with the observation of Andes-Koback and Keating^19^ that these vesicles can split into daughter cells that contain either the PEG or the dextran solution. After a volume reduction of 35%, we continued with a 5 μs steady-state simulation. A detailed shape analysis (Figure S2) revealed that despite the slow pumping rate of 0.2%, the vesicle shape shifted toward an even more prolate state while perfectly maintaining the volume.

### Membrane Proteins

**Figure 5:**
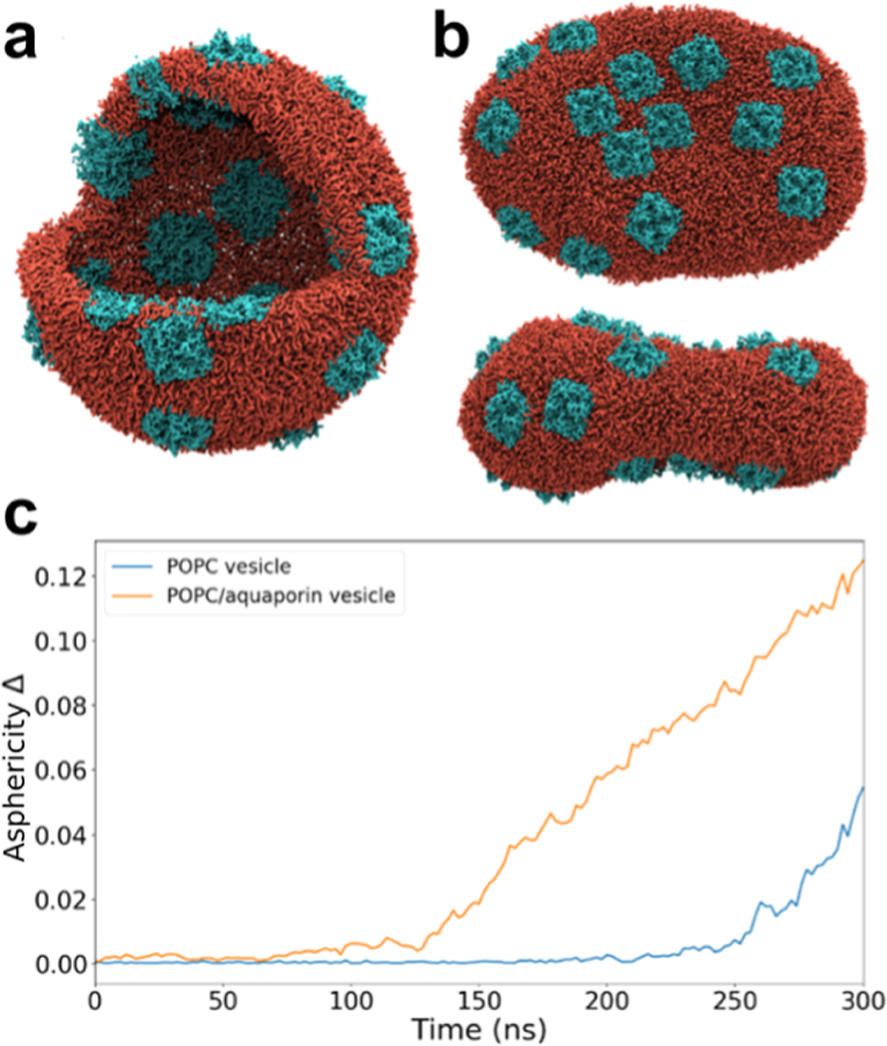
Hypertonic osmotic shock applied to a POPC vesicle with and without transmembrane proteins. Snapshots show (a) the initial spherical vesicle with 25 aquaporins (cyan) embedded in a POPC membrane (red) and (b) the top and side views of the vesicle after a 60% volume reduction. (c) Shape deformation is quantified using the asphericity analysis (described in Supporting Information).

So far, we have considered only pure lipid membranes in our test cases. With the next system, we want to show that Shocker can also be applied to vesicles with a more complex membrane composition, including proteins. To this end, we constructed a CG POPC vesicle with a radius of 16 nm, including 25 aquaporin proteins (Figure 5a). The water-conducting channel of aquaporin is closed, and no counterflow of water occurs. Again, the simulation was performed with a pumping rate of 200 particles every 2 ns, corresponding to 0.2% of the initial vesicle volume. For reference, we performed the same simulation for a pure POPC vesicle.

After a volume reduction of 60%, the initially spherical vesicle transformed into an oblate shape with the aquaporins mostly situated on the flat surfaces, as can be seen in the top and side views in Figure 5b and Movie S3. The areas with a strong curvature contained little to no proteins.

To quantify the shape changes of the vesicles over the course of the simulation, we computed the asphericity parameter Δ (see Supporting Information Methods). An asphericity value of 0 corresponds to a perfect sphere, whereas a value above zero indicates a deviation from a spherical shape. The asphericity analysis in Figure 5c shows that the initially spherical vesicle (asphericity parameter close to zero) experiences a sudden shape change at around 125 ns in the presence of aquaporin, followed by a steady increase in asphericity. In the case of a POPC vesicle without proteins, however, the shape transformation develops more gradually. During a 5 μs steady-state simulation, the final vesicle of the pumping simulation turned slightly more oblate following the movement of aquaporins toward the flat surfaces of the vesicle. To quantify this, we calculated the oblicity parameter *S* (Figure S3). The oblicity parameter *S* becomes increasingly negative, confirming the shift toward an even more oblate-shaped vesicle.

### Atomistic Osmotic Shock

Lastly, we demonstrate that Shocker is compatible not only with CG but also with AA systems. To reduce the computational costs, we chose a double bilayer system instead of a vesicle. The system consists of two POPC bilayers in a 10 × 10 × 25 nm^3^ box, initially positioned with an 8 nm gap between the two membranes. A hypertonic osmotic shock with a pumping rate of 50 water molecules every 2 ns was performed, corresponding to relocating 0.16% of the volume of the central compartment during each cycle.

**Figure 6:**
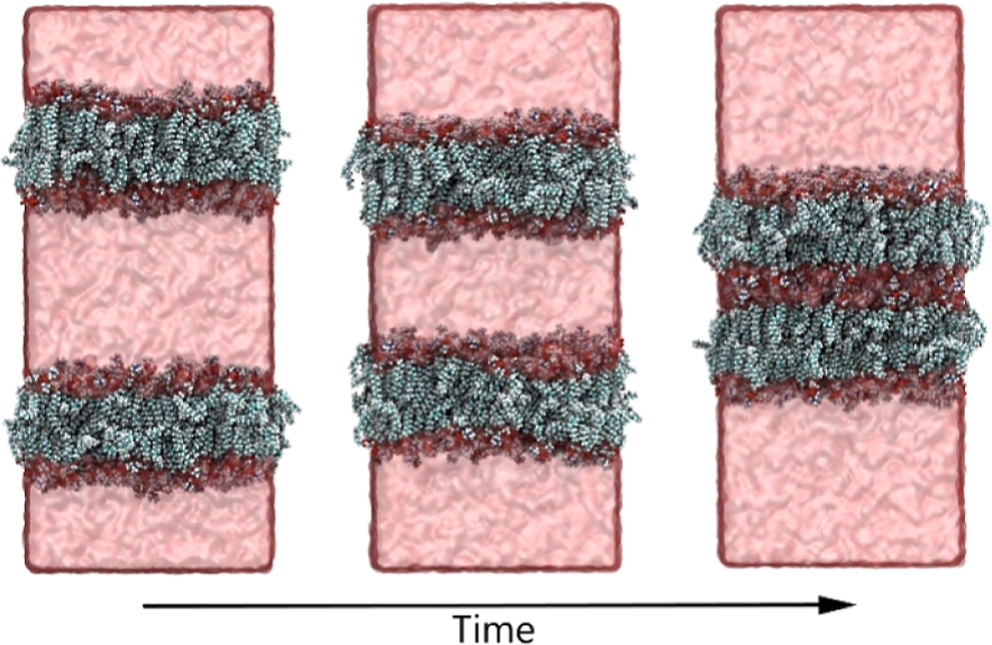
Hypertonic osmotic shock applied to an atomistic double bilayer system. Water is relocated from the central compartment between the bilayers to the space outside the bilayers (crossing the periodic boundaries). Over time, the membranes gradually move closer together.

Figure 6 and Movie S4 show that over the course of the pumping simulation, the volume of the central water compartment between both membranes decreases until the point that both membranes almost touch. At this point, there is still water between the membranes, but Shocker will not relocate those. In order to maintain the hydration shell, water molecules located in membrane bins are excluded from the pumping procedure.

### Performance

**Figure 7:**
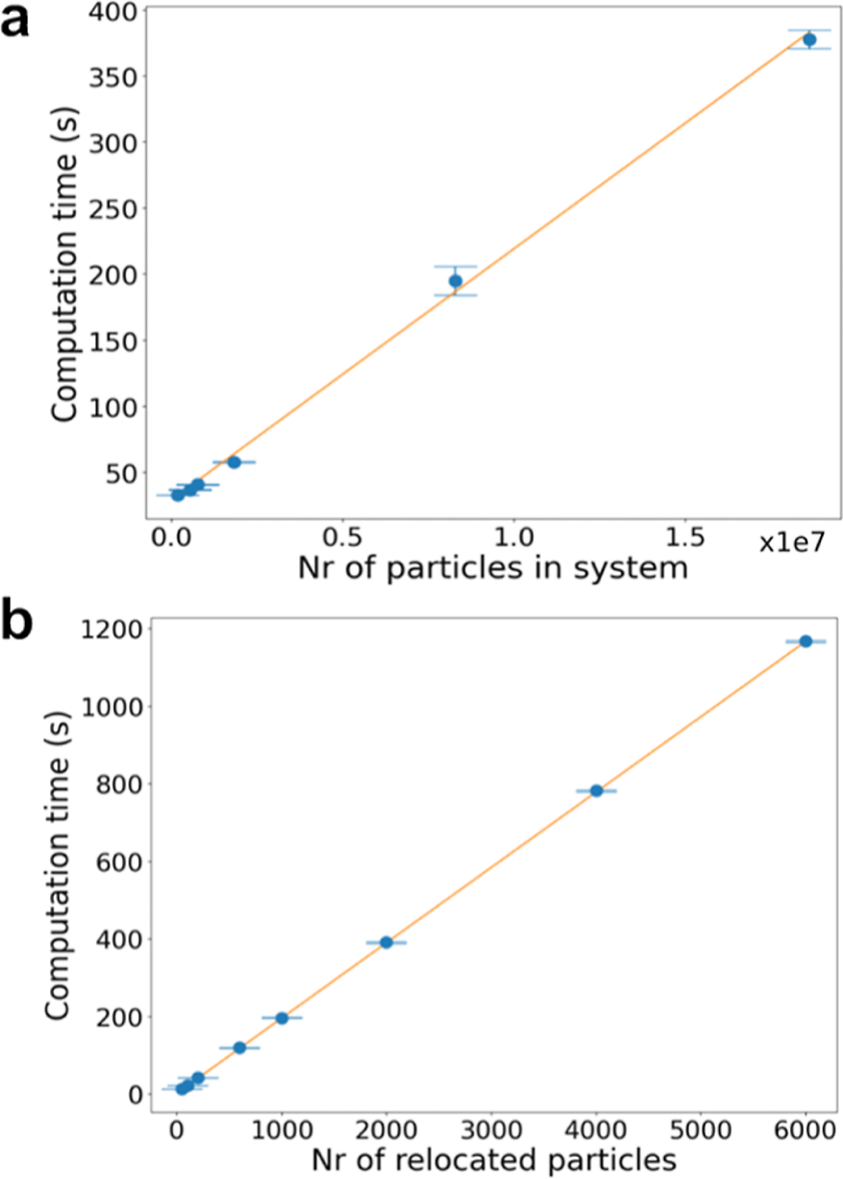
Computation time of a single pumping cycle. The computation time of a single pumping cycle is measured for (a) different system sizes when relocating 200 particles and for (b) different numbers of particles to be relocated in a fixed system size of 750,000 particles. Error bars indicate the standard deviations obtained from 10 replicas.

To benchmark the performance of Shocker, we measured the time needed to complete one pumping cycle. This was done for various test cases with different system sizes ranging from 152,000 to 18,000,000 CG particles, where we relocated 200 solvent particles. Subsequently, we performed tests in which the total number of particles was kept fixed at 750,000, while the number of relocated particles ranged from 100 to 6000. In both cases, a bin-edge size of 1.3 nm was used. All test systems were run on the same Ubuntu desktop computer containing an Intel Xeon CPU (6 cores, hyperthreading, 4.5 GHz) and 16GB of DDR4 memory. The results of these test runs are collected in Figure 7.

Figure 7a shows that there is a linear relationship between the time Shocker needs to perform one pumping cycle and the number of particles of which the system consists. When relocating 200 particles, Shocker is able to complete one pumping cycle within 6 min for all tested system sizes. Figure 7b shows that a linear relationship also exists between the number of relocations and the computation time for one cycle. For a system size of 750,000 CG particles, which is around the size of most of the systems in this study, Shocker needs 42 s to perform one relocation cycle. However, when relocating 6000 particles, which is almost 1% of the content of the box, Shocker needs 20 min of computation time. Considering that for a system of this size, a single 2 ns production run takes 18 min even this relocation time is acceptable.

## Discussion and Conclusions

Osmosis is a fundamental process that is coupled to many processes at the cellular level.^40^ Beyond being a simple environmental factor, osmotic shocks are also frequently used as a tool in synthetic chemical biology to, for example, manipulate the membrane shapes of cells.^11,41^ Despite its widespread occurrence in biology and synthetic biology, a molecular-level picture of how membrane shape deformations are steered by osmosis remains hidden from experimental techniques and still poses many open questions. To answer those questions and elucidate the molecular driving forces, simulation approaches such as coarse-grained molecular dynamics simulations are commonly employed.^23,42,43^ Since simulating an osmotic shock explicitly in MD is a time-consuming process, researchers circumvent it by either using pre-deformed vesicles^26^ or removing solvent molecules from the interior in the initial setup.^24^ Even though these studies can give valuable insights into vesicle shape transformations, including budding, fusion, and fission, they allow very little control over the final membrane shapes or the deformation pathways. To allow the efficient and controlled simulation of osmotic shocks in MD using explicit water models, we presented and benchmarked the Shocker Python program.

### Robust Simulation Protocol

Shocker mimics an osmotic shock by moving solvent particles between compartments, according to the direction of the osmotic shock, during several pumping cycles. A pumping cycle starts by identifying an inner and outer solvent compartment by using an efficient binning algorithm. Subsequently, a number of solvent molecules defined by the pumping rate are moved to the other compartment, depending on the type of osmotic shock (hypertonic or hypotonic). Instead of just randomly placing solvent molecules, it is taken care that the system is perturbed as little as possible by finding the largest gap within a solvent bin. Finally, a short equilibration is performed before a new cycle begins. We note that the program not only implements hypertonic and hypotonic shocks but also is capable of performing a steady-state simulation that keeps the number of solvent molecules constant by counteracting the natural solvent permeation.

To assess the extent of perturbation due to solvent relocation, we conducted two benchmark osmotic shocks utilizing a slow and fast pumping rate. The test system consisted of a water box at the all-atom and CG Martini levels, in which either 0.05 or 1% of water molecules were relocated within the water box. The distance distribution between the newly placed water particles and all other water particles in Figure S4 showed that the method produces no direct overlaps, even for the fast pumping rate. This conclusion was further supported by the fact that the simulation can be continued after relocating the water particles without the need for energy minimization. While the relocation does produce a sudden increase in potential energy, this energy is quickly dissipated (see Figure S5). We note that, in general, within the Shocker protocol, velocities are preserved after water relocation, even for the more complex test cases. However, especially in crowded systems, water molecules may have to be removed from the immediate solvation shell of other molecules, such as proteins and polymers, in solution. To ensure a stable simulation protocol, Shocker offers the possibility to automatically add energy minimization and equilibration steps into the pipeline, even though for most systems they are not required. Only the crowded polymer-filled vesicles required such additional measures from the test cases surveyed. Additionally, as a built-in safety net against numerical failure of simulations, Shocker returns to a state before the error occurs and tries to relocate other water particles to new positions. This continued until the production run finished correctly.

### Realistic Membrane Shapes

To assess whether Shocker produces realistic membrane shapes, we tested the protocol on increasingly complex membrane systems. First, we applied a hypertonic osmotic shock to a simple POPC vesicle and examined the effect of different solvent pumping rates on its shape. To monitor the shapes over time, we constructed a shape phase diagram, which clearly showed two distinct shape pathways generally obeying the experimentally determined shape boundaries.^38^ Slower pumping rates result in more prolate shapes as opposed to faster pumping rates, which yield oblate shapes. This finding is consistent with an earlier simulation study.^30^ After the target volume was reached, a 5 μs steady-state simulation was performed on both vesicles. While the interior vesicle volume was kept constant, membrane shape changes could be observed. Slower pumping rates yield more stable shapes and are closer to equilibrium shape deformations, while the vesicle subjected to a faster pumping rate experienced a significant shift in shape over time. This example demonstrates that the user must carefully choose an appropriate pumping rate. We recommend 0.2% as a good compromise between being fast while allowing sufficient relaxation time to avoid kinetic artifacts.

By applying a hypotonic osmotic shock to a POPC vesicle, we demonstrated that our protocol can be used to inflate a vesicle up to the point of rupture. Furthermore, after rupture, the program automatically switches to a regular no-pumping simulation, allowing the vesicle to heal. An interesting result was the smaller radius and volume as well as the larger membrane thickness of the healed vesicle compared to those of the initial vesicle. For this observation, we consider two possible explanations. On the one hand, it is possible that the initial vesicle is not completely tensionless. In this case, the vesicle initially contains a larger amount of water than is energetically favorable and/or has an imbalance of lipids between the inner and outer vesicle leaflets. This can be corrected by the efflux of water after rupture and by an accelerated lipid flip-flop at the large pore rim, which results in a vesicle with a radius slightly smaller than the initial one. On the other hand, with the rapid opening followed by a fast healing process, more water might be excreted than inserted during the osmotic shock. This kinetic effect would also result in a vesicle with a volume smaller than that of the initial one. While further analysis is needed to confirm either hypothesis, we have no reason to believe the observed result is an artifact of the osmotic shocking protocol.

To further increase the complexity, we considered a system consisting of a phase-separated membrane with an aqueous two-phase polymer system on the interior. Previously, similar systems have been shown to undergo asymmetric fission in experiments under osmotic shock.^19^ While we did not see fission, the system deformed from a spherical vesicle to a prefission, dumbbell state. The polymer interior remains phase-separated and aligns with the neck at the interface of the two lipid phases. This behavior is consistent with the experimental results observed for similar systems.^19,44,45^

Next, we tested membrane deformations in the presence of proteins. It is well known that proteins induce membrane curvature or have an intrinsic curvature preference.^46^ Given the crowdedness of realistic cell membranes, it is of paramount importance to capture shape deformations in the presence of proteins. Our test system consisted of a vesicle with 25 aquaporins embedded in a POPC membrane. Under a hypertonic shock with a volume reduction of 40%, the membrane adopts a flat disk shape. The strongest curvature occurs in the areas with the lowest protein density. The difference in shape development between a pure POPC vesicle and the protein-containing vesicle must therefore originate from the proteins embedded in the membrane, which rigidify the membrane. A nonspherical shape can alleviate the energetic cost of bending such a stiff membrane, in particular as the system can form protein-rich and protein-poor domains. The vesicle, therefore, remains spherical until a point where the low internal pressure makes the vesicle collapse into a flat disk shape. Hereby, the strong curvature appears in the most flexible membrane areas, i.e., those with the lowest protein density. The early deformation in the protein-containing vesicle can be explained by the fact that these membrane proteins prefer a flat membrane surface and, thus, promote an early deformation to create flat surfaces. This phenomenon became even more clear when subjecting the final vesicle to a 5 μs steady-state simulation, where the proteins moved to flat membrane areas, resulting in a more oblate-shaped vesicle. Given that aquaporin has previously been reported to prefer zero-curvature regions on the membrane,^47^ these results validate the applicability of our protocol to membranes containing proteins.

Lastly, we tested Shocker on an AA system consisting of two flat POPC bilayers. A hypertonic shock was performed, therefore reducing the volume of the compartment between the bilayers. As expected, the bilayers moved closer together, consistent with a hypertonic shock.

### Limitations and Future Extensions

To measure the performance of the water relocation code, we benchmarked systems ranging from 150,000 to 18 million particles. On a desktop computer, a single pumping cycle is completed within minutes when relocating 200 particles. Generally, the relocation is, therefore, much faster than any equilibration or production run on large systems. Hence, we consider Shocker to be performant enough, even for the largest currently studied systems. In principle, the speed of water relocation can even be increased by increasing the number of water particles. However, as mentioned earlier, this can influence the shape deformation pathways and potentially cause numerical stability problems.

An important current limitation of the tool is that Shocker works with only two water compartments. Even though this is sufficient for applications to vesicles, tubes, and double bilayer systems, many higher-order membrane shapes of interest, such as multilamellar vesicles, exist. The principal limitation of the two compartments results from the binning procedure used to identify the compartments. Combining Shocker with more advanced protocols, such as the MDVoxel Segmentation protocol,^48^ could be a future extension to make such systems possible.

Finally, we note that Shocker implements some common shape analysis metrics that can be used to characterize the vesicle shapes along the pumping simulations. In order to compute properties such as the area and volume of the deformed vesicles, first, any periodic boundary crossings need to be removed to generate the continuous surface required in the second step. The surface of each leaflet is then generated using the PyVista surface reconstruction method^49^ which allows an estimation of the vesicle area and volume. Removing periodic boundary crossings using the in-house method, however, can fail if the object crosses the PBC multiple times. If the reconstruction fails, no continuous surface can be generated, which ultimately leads to the failure of the analysis. Using more advanced algorithms such as MDVWhole^50^ would be an option to make this step more robust and ensure the analysis protocol works for shapes split across the PBC in arbitrary ways. The shape reconstruction can also fail when the membrane displays regions of high curvature, where the separation between leaflets is so small that the surfaces cannot be reconstructed properly by the used algorithm. Further surface refinement might be required to proceed with the analysis.

We conclude that the Shocker Python program outlined in this paper offers a robust protocol to study the effect of osmotic shocks on membranes in a realistic and direct manner.

## Methods

### System Setup

All CG vesicle systems used in this work were constructed using TS2CG,^51^ an MD tool designed to generate initial configurations for lipid membrane structures with user-defined compositions (including proteins) and shapes. Lipid parameters were taken from the recently published Martini 3^36^ force field. The topology and structure files of CG proteins were generated with Martinize2^52^ using the Martini 3^36^ force field. Elastic bonds were applied to aquaporin (PBD ID: 1J4N(53)) to maintain the secondary structure as well as the tetrameric assembly. To fill the interior of those vesicles with polymers, Polyply^54^ was used, a Python suite designed for generating input files and system coordinates for macromolecules. Topologies for PEG and dextran were taken from Polyply^54^ and our recently published Martini 3 extension for carbohydrates,^55^ respectively. A development version of the recently published cholesterol model^56^ was used. System solvation and placement of sodium chloride ions (if needed) were done using the solvation script provided by TS2CG.^51^

The AA double bilayer system was constructed in two steps: First, we generated a single POPC bilayer using the CHARMM-GUI^57^ with CHARMM36 force field^37^ parameters and TIP3P water. Subsequently, Gromacs tools were used to duplicate the entire system in the *z*-dimension.

### System Details

Table S1 displays the system size and composition of all of the setups used in this work. The lipid names are consistent with the names used in the Martini CG lipidomics database (http://cgmartini.nl/index.php/force-field-parameters/lipids). The lipid ratio in Table S1 is determined by TS2CG^51^ during lipid placement.

After placing the lipids and initial energy minimization using the steepest descent algorithm, a series of equilibration runs with different membrane pore sizes was performed (as described in a previous work^58^). This allows lipid redistribution between the two leaflets and equilibration of the solvent content between the interior and exterior solvent compartments. The pores were gradually closed during the equilibration before the production run.

### Simulation Parameters

All simulations were executed with GROMACS version 2021.2.^59^ Simulation parameters for CG simulations were based on standard values used in the Martini benchmarking paper.^60^ CG simulations were performed in the *NPT* ensemble at a constant pressure and temperature of 1 bar and 310 K, respectively, except for the simulation with the polymer-filled vesicle, which was performed at a temperature of 323 K. The pressure and temperature were maintained using the anisotropic Berendsen coupling^61^ and velocity rescaling thermostat,^62^ respectively. Furthermore, for production runs, an integration time step of 20 fs was chosen using a leapfrog algorithm for integrating the equations of motion.

For the AA simulation, the pressure of 1 bar and temperature of 310 K were maintained using the Parrinello–Rahman pressure coupling^63^ and the Nose–Hoover thermostat,^64^ respectively. An integration step of 2 fs was used.

### Analysis Details

All videos and snapshots of the simulations were rendered with VMD.^65^ Calculation of the volume, area, and asphericity parameters of vesicles is an intrinsic feature of Shocker, as explained in more detail in the Supporting Information.

### Use and Availability

Shocker can be found in our GitHub repository (https://github.com/marrink-lab/shocker) and installed according to the README file. It is accompanied by several tutorials and data sets to perform hypertonic and hypotonic osmotic shock simulations. The initial configurations of all experiments described in this paper and the corresponding command-line options to invoke Shocker can be found on the GitHub page.

## Supporting Information Available

Vesicle shape analysis after 5 μs of steady-state simulation for a POPC vesicle under different pumping rates and a phase-separated, polymer-filled vesicle; oblicity analysis for an aquaporin-containing vesicle during 5 μs of steady-state simulation; assessment of system perturbation during water relocation; overview of performed simulations; and supplementary methods describing Shocker’s built-in analysis features used in the presented example cases (PDF)
Movie S1 - Hypotonic osmotic shock on a POPC vesicle. External water molecules are omitted for clarity. Water molecules in the vesicle’s interior are shown as green spheres, which slowly start to diffuse through the lipid bilayer. Once the membrane ruptures, the excess water molecules are expelled from the vesicle (MP4)
Movie S2 - Deformation of a phase-separated, polymer-filled vesicle under hypertonic osmotic shock. The vesicle deforms into a dumbbell shape, with the interfacial region of both the lipid and polymer phases aligned in the neck of the dumbbell (MP4)
Movie S3 - Deformation of a POPC vesicle with 25 aquaporin proteins embedded into the membrane under hypertonic osmotic shock. The vesicle deforms into an oblate shape with most aquaporins residing on the flat surfaces of the oblate (MP4)
Movie S4 - Osmotic shock applied to an atomistic double-bilayer system. Water is relocated from the central compartment to the other one. Over time, the central compartment shrinks, and the two bilayers start approaching each other (MP4)
