# Supplementary material for: Shocker—A Molecular Dynamics Protocol and Tool for Accelerating and Analyzing the Effects of Osmotic Shocks
Source: J Chem Theory Comput. 2023 Dec 18;20(1):212–23. doi: 10.1021/acs.jctc.3c00961 (PMC10782443; doi:10.1021/acs.jctc.3c00961)
Supplement: Supplementary file 1 — ct3c00961_si_001.pdf [file ct3c00961_si_001.pdf]

Supporting Information:

Shocker - a molecular dynamics protocol and tool for accelerating and analyzing the effects of osmotic shocks

Marco P. A. van Tilburg[1], Siewert J. Marrink[1], Melanie König[1†], Fabian Grünewald[1,2†]

[1] Groningen Biomolecular Sciences and Biotechnology Institute and Zernike Institute for Advanced Materials, University of Groningen, 9747 AG Groningen, The Netherlands

[2] Heidelberg Institute for Theoretical Studies (HITS), Schloss-Wolfsbrunnenweg 35, 69118 Heidelberg, Germany

† corresponding authors: m.konig@rug.nl, Fabian.Gruenewald@h-its.org

Supporting Figures

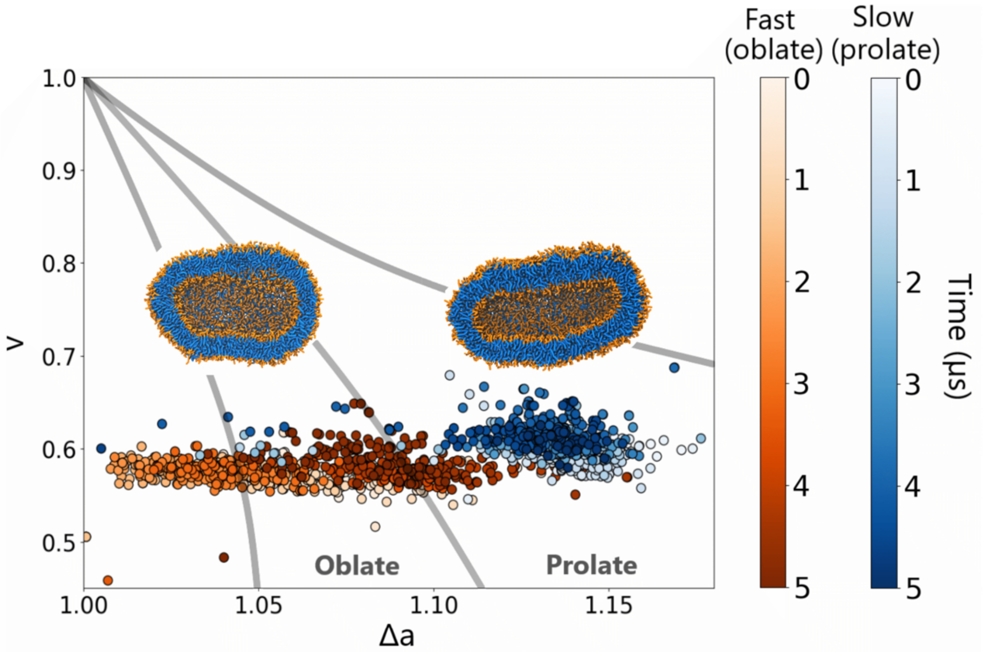

Figure S1 Shape analysis of steady-state simulations of different pumping rates. A hypertonic shock was performed on POPC vesicles using pumping rates of 0.1% (slow) and 1% (fast) of the initial interior vesicle volume. After a 40% volume reduction, a 5 µs steady-state simulation was launched and the changes in shape for the two pumping rates are indicated. Each dot in the shape diagram represents the vesicle shape after 20 ns, corresponding to 10 evaluations of the vesicle volume. The color indicates the temporal evolution for the two pumping rates (white to dark red/blue). The snapshots of cut vesicles on the left and right show the final shape of the steady-state simulation for the fast and slow pumping rate, respectively.

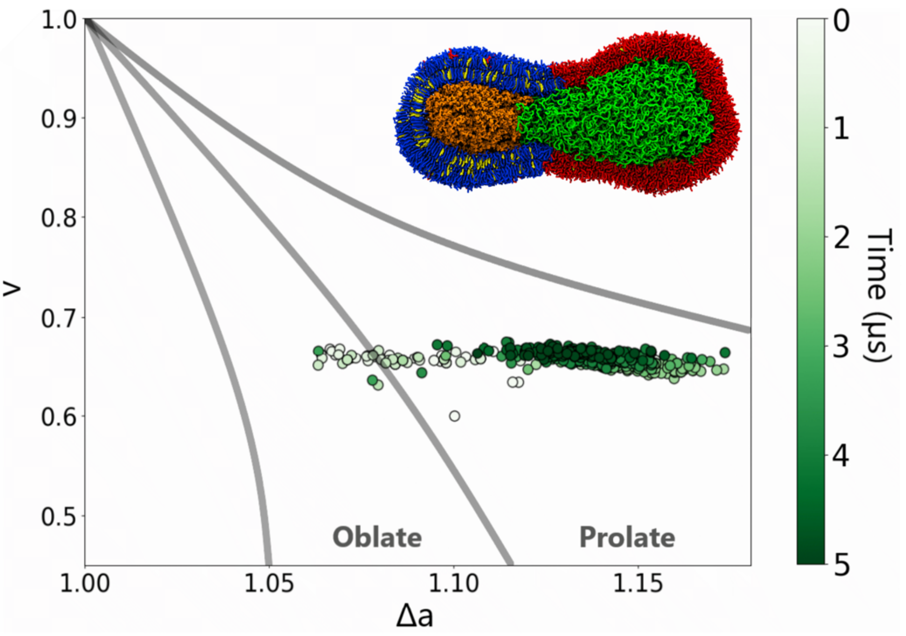

Figure S2 Shape analysis of steady-state simulations for polymer-filled phase-separated vesicle. A hypertonic shock was performed on a vesicle with a phase-separated DPPC/CHOL/DFPC membrane and filled with dextran and PEG. After a 35% volume reduction of the initial enclosed water volume, a 5 µs steady-state simulation was launched and the changes in shape are indicated. The color indicates the temporal evolution from white to dark green. The snapshot shows the equilibrated vesicle shape after 5 µs of steady-state simulation.

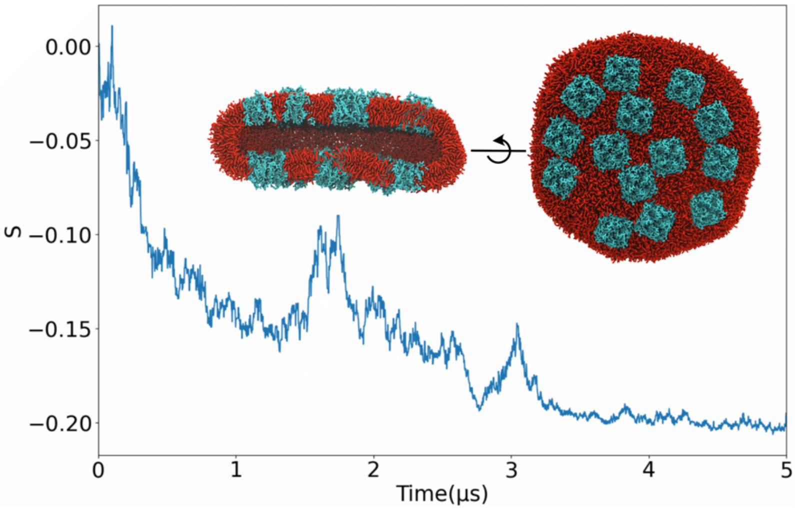

Figure S3 Oblicity analysis of steady-state simulations with and without transmembrane proteins. A hypertonic shock was performed on a POPC vesicle with and without aquaporin embedded into the membrane. After a 40% volume reduction, a 5 µs steady-state simulation was launched and the changes in oblicity S are calculated as described in the Supplementary Methods. A negative oblicity value indicates an oblate shape. The snapshots show the equilibrated vesicle shape after 5 µs of steady-state simulation from the side (cut) and top.

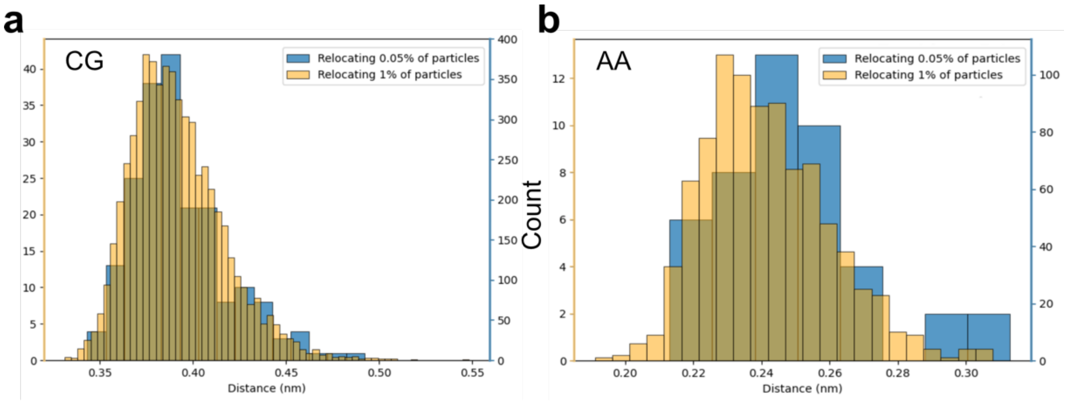

Figure S4 Histograms of water-water distances after relocation. The center of mass distances of all relocated water particles to the closest other water particles in the bin for (a) a CG system and (b) an AA system. We applied pumping rates of 0.05% (yellow) and 1% (blue) for both systems, corresponding to 300 and 6000 water beads for the CG system or 30 and 600 water molecules for the AA system. The setup is a water box in which particles are moved from one side to the other.

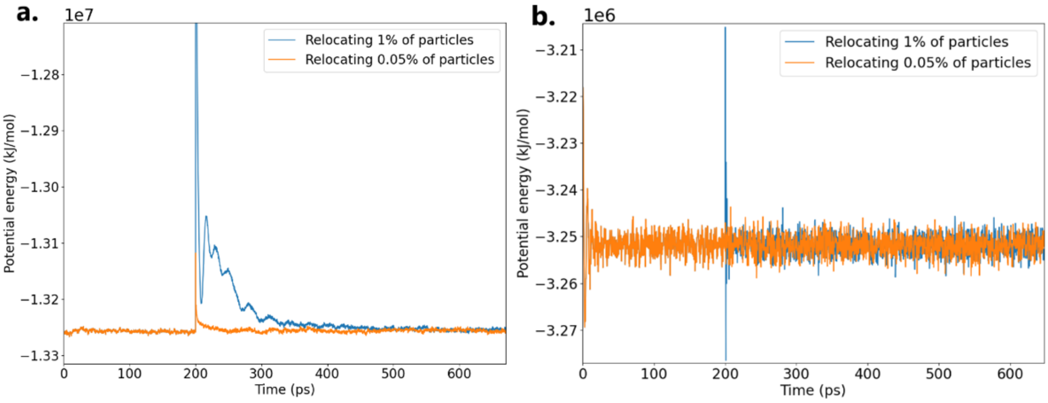

Figure S5 Evolution of potential energy during a pumping cycle. After 200 ps simulation time, water particles are moved for (a) a CG system and (b) an AA system as described in **Figure S4**.

Supporting Tables

Table S1 System composition and simulation details for all example systems. Shocker was used to perform hypertonic and hypotonic osmotic shock simulations for a variety of different systems. Simulation times varied between 200 ns and 2400 ns depending on the pumping rate.

	Example system	Total no.of particles	Membrane composition	Lipid ratio (outer/inner)	Vesicle radius (nm)	Pumping rate (per 2 ns)	Initial interior volume (no. of solvent particles)
CG MARTINI	Test pumping rate (hypertonic)	184,016	POPC	1797/603	7.5	Slow: 10<br>Fast: 100	10,000
	Exploding vesicle (hypotonic)	1,843,883	POPC	4892/3492[a]<br>4769/3615[b]	15.9	200	120,000
	Polymer vesicle (hypertonic)	770,237	DPPC/CHOL/DFPC (0.30/0.20/0.50)	5267/3251	16.4	200	94,000
	Protein vesicle (hypertonic)	1,816,104	POPC with 25 aquaporin (PDB-ID: 1J4N)	3722/2635	15.7	200	110,000
	POPC vesicle (hypertonic)	1,843,883	POPC	4892/3492	15.9	200	120,000
AA	Double bilayer	230,784	POPC	300 lipids per bilayer	–	40	25,000

[a] initial configuration
[b] after pore closure

Supporting Methods

Analysis features of Shocker. The Shocker package has a few in-built analysis methods, which, if enabled, store useful properties of a vesicle each pumping cycle. In the first cycle, the inner and the outer membrane area as well as the vesicle volume are calculated. It is verified if the structure is whole or if it crossed the periodic boundaries of the simulation box. In the latter case, the smaller pieces are transferred to their original positions to make the structure whole again before further calculations. Subsequently, a point cloud is generated based on the lipid linker positions, in the case of Martini force field only the GL1 beads are considered. Alternatively, the user can provide the desired atom type for analysis. Subsequently, the Python library pyvista [1] was used to generate a triangulated surface of which the area and volume can be determined. This method allows for accurately calculating the area and volume of even the most irregularly shaped vesicles.

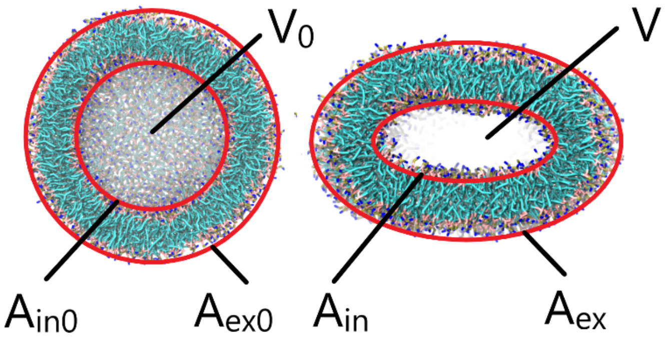

Figure S6 Schematic representation of the different vesicle shape properties analyzed by Shocker. When a pumping simulation is initialized (left), Shocker determines the initial interior volume (V_0), the area of the inner leaflet (A_{in0}) as well as the area of the outer leaflet (A_{ex0}). At the end of each pumping cycle (right), the current volume (V), the area of the inner leaflet (A_{in}), and the area of the outer leaflet (A_{ex}) are measured. With these values, Shocker can calculate the relative volume (v) and reduced area difference (Δa) which is used to describe shape changes of vesicles.

In case solutes are present in the simulation box, it is possible to calculate the solute concentration inside and outside the vesicle. For this purpose, the vesicle volume from the previous step and the number of solute molecules are determined. The earlier calculated clusters are used to count the solutes in the inner and outer compartments.

For each pumping cycle, Shocker calculates the area of the inner leaflet (A_{in}), the area of the outer leaflet (A_{ex}), and the interior volume (V) to calculate the relative volume v and the reduced area difference Δa (see **Figure S6**). Hereby, relative volume v is defined as

$$v = \frac{V_0}{V} \qquad \text{Eq. 1}$$

and the reduced area difference Δa as

$$\Delta a = \frac{\Delta A}{\Delta A_0} = \frac{A_{ex} - A_{in}}{A_{ex0} - A_{in0}} \qquad \text{Eq. 2}$$

where V_0, A_{in0} and A_{ex0} denote to the volume, area of the inner and outer leaflet at the beginning of the pumping simulation. A combination of these parameters quantifies the shape of a vesicle of the course of the pumping simulation.[2]

Lastly, Shocker can determine the asphericity parameter Δ and oblicity parameter S. These values indicate the degree of asphericity and oblicity, respectively, of a set of points [3] and are calculated according to the eigenvalues of the gyration tensor

$$T_{\alpha\beta} = \frac{1}{2N^2}\sum_{i,j=1}^{N}(r_{i\alpha} - r_{j\alpha})(r_{i\beta} - r_{j\beta}) \qquad \text{Eq. 3}$$

with N being the total number of particles and $r_{i\alpha}$ is the α-th component of the position of particle i and α and β = x,y,z. The eigenvalues of T, denoted by λ_i, are the squares of the three principal radii of gyration. Thus, $R_g^2 = trT = \sum_{i=1}^{3}\lambda_i$.

The asphericity parameter Δ and oblicity parameter S are defined as

$$\Delta = \frac{3}{2}\frac{[\sum_{i=1}^{3}(\lambda_i - \bar{\lambda})^2]}{(trT)^2} \qquad \text{Eq. 4}$$

And

$$S = \frac{27[\prod_{i=1}^{3}(\lambda_i - \bar{\lambda})]}{(trT)^3} \qquad \text{Eq. 5}$$

where $\bar{\lambda} = \frac{trT}{3}$ is the average eigenvalue of the gyration tensor. The asphericity parameter Δ is 0 for a perfectly spherical set of points, while S is positive in the case of a prolate shape and negative for an oblate shape.

Supporting References

(1) Sullivan, C.; Kaszynski, A. PyVista: 3D plotting and mesh analysis through a streamlined interface for the Visualization Toolkit (VTK). *Journal of Open Source Software* **2019**, *4* (37), 1450.
(2) Käs, J.; Sackmann, E. Shape transitions and shape stability of giant phospholipid vesicles in pure water induced by area-to-volume changes. *Biophysical Journal* **1991**, *60*, 825-844.
(3) Dima, R. I.; Thirumalai, D. Asymmetry in the shapes of folded and denatured states of proteins. *The Journal of Physical Chemistry B* **2004**, *108* (21), 6564-6570.
